# Supplementary material for: Identification of photoperiod-induced specific miRNAs in the adrenal glands of Sunite sheep (Ovis aries)
Source: Front Vet Sci. 2022 Jul 22;9:888207. doi: 10.3389/fvets.2022.888207 (PMC9354845; doi:10.3389/fvets.2022.888207)
Supplement: Supplementary file 1 [file Data_Sheet_1.ZIP › Supplementary file/miRDeep2 file.zip!/miRDeep2 file/Know_Structure/LP42A1/oar-mir-181a-2.pdf]

miRBase precursor : oar-mir-181a-2
Total read count : 23238
oar-miR-181a read count : 23087
remaining reads : 151

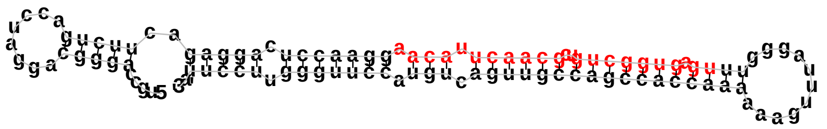

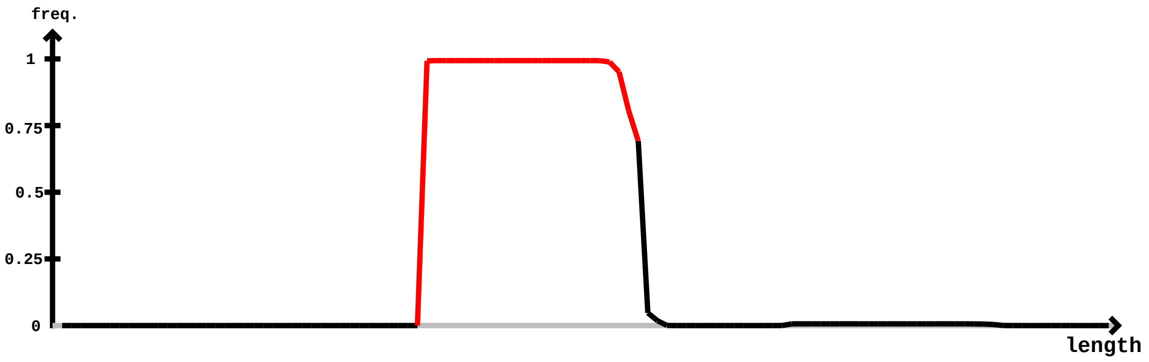

oar-miR-181a

```
5'- ugccagggcaggauccagucuucagaggacuccaaggaacauucaacgcugucggugaguuugggauuugaaaaaaccaccgaccguugacuguaccuuggguuccuua   -3'    exp
    ....(((((........)))))..(((((.(((((((.(((.((((((..(((((((.((((...........))))))))))))))))).))).))))))).))))).   reads     mm     sample
    .....................................aacauucaacgcugucgg......................................................   4         0      seq
    .....................................aacauucaacgcugucggu.....................................................   115       0      seq
    .....................................aacauucaacgcugucggug....................................................   858       0      seq
    .....................................aacauucaacgcugucgguga...................................................   3381      0      seq
    .....................................aacauucaacgcugucggugag..................................................   2653      0      seq
    .....................................aacauucaacgcugucggugagu.................................................   14955     0      seq
    .....................................aacauucaacgcugucggugaguu................................................   682       0      seq
    .....................................aacauucaacgcugucggugaguuu...............................................   412       0      seq
    .....................................aacauucaacgcugucggugaguuug..............................................   6         0      seq
    ......................................acauucaacgcugucggug....................................................   2         0      seq
    ......................................acauucaacgcugucgguga...................................................   1         0      seq
    ......................................acauucaacgcugucggugag..................................................   2         0      seq
    ......................................acauucaacgcugucggugagu.................................................   11        0      seq
    ......................................acauucaacgcugucggugaguu................................................   1         0      seq
    ......................................acauucaacgcugucggugaguuu...............................................   1         0      seq
    .......................................cauucaacgcugucggugagu.................................................   1         0      seq
    .......................................cauucaacgcugucggugaguuu...............................................   1         0      seq
    .........................................uucaacgcugucggugagu.................................................   1         0      seq
    ..........................................................................aaccaccgaccguugacuguac.............   5         0      seq
    ...........................................................................accaccgaccguugacugu...............   6         0      seq
    ...........................................................................accaccgaccguugacugua..............   13        0      seq
    ...........................................................................accaccgaccguugacuguac.............   32        0      seq
    ...........................................................................accaccgaccguugacuguacc............   88        0      seq
    ...........................................................................accaccgaccguugacuguaccu...........   6         0      seq
    ...........................................................................accaccgaccguugacuguaccuu..........   1         0      seq
```
